# Considerations for artificial intelligence clinical impact in oncologic imaging: an AI4HI position paper

**DOI:** 10.1186/s13244-022-01220-9

**Published:** 2022-05-10

**Authors:** Luis Marti-Bonmati, Dow-Mu Koh, Katrine Riklund, Maciej Bobowicz, Yiannis Roussakis, Joan C. Vilanova, Jurgen J. Fütterer, Jordi Rimola, Pedro Mallol, Gloria Ribas, Ana Miguel, Manolis Tsiknakis, Karim Lekadir, Gianna Tsakou

**Affiliations:** 1 Radiology Department and Biomedical Imaging Research Group (GIBI230), La Fe Polytechnics and University Hospital and Health Research Institute, Valencia, Spain; 2 Department of Radiology, Royal Marsden Hospital and Division of Radiotherapy and Imaging, Institute of Cancer Research, London, UK; 3 Department of Radiology, The Royal Marsden NHS Trust, London, UK; 4 Department of Radiation Sciences, Diagnostic Radiology, Umeå University, 901 85 Umeå, Sweden; 5 2nd Department of Radiology, Medical University of Gdansk, 17 Smoluchowskiego Str, 80-214 Gdansk, Poland; 6 Department of Medical Physics, German Oncology Center, 4108 Limassol, Cyprus; 7 Department of Radiology, Clínica Girona, Institute of Diagnostic Imaging (IDI)-Girona, Faculty of Medicine, University of Girona, Girona, Spain; 8 Department of Radiology and Nuclear Medicine, Radboud University Medical Center, Nijmegen, The Netherlands; 9 CIBERehd, Barcelona Clinic Liver Cancer (BCLC) Group, Department of Radiology, Hospital Clínic, University of Barcelona, Barcelona, Spain; 10 Foundation for Research and Technology Hellas, Institute of Computer Science, Computational Biomedicine Lab (CBML), FORTH-ICS Heraklion, Crete, Greece; 11 Departament de Matemàtiques and Informàtica, Artificial Intelligence in Medicine Lab (BCN-AIM), Universitat de Barcelona, Barcelona, Spain; 12 Maggioli S.P.A., Research and Development Lab, Athens, Greece

**Keywords:** Artificial intelligence, Oncologic imaging, Prediction models, Clinical validation

## Abstract

To achieve clinical impact in daily oncological practice, emerging AI-based cancer imaging research needs to have clearly defined medical focus, AI methods, and outcomes to be estimated. AI-supported cancer imaging should predict major relevant clinical endpoints, aiming to extract associations and draw inferences in a fair, robust, and trustworthy way. AI-assisted solutions as medical devices, developed using multicenter heterogeneous datasets, should be targeted to have an impact on the clinical care pathway. When designing an AI-based research study in oncologic imaging, ensuring clinical impact in AI solutions requires careful consideration of key aspects, including target population selection, sample size definition, standards, and common data elements utilization, balanced dataset splitting, appropriate validation methodology, adequate ground truth, and careful selection of clinical endpoints. Endpoints may be pathology hallmarks, disease behavior, treatment response, or patient prognosis. Ensuring ethical, safety, and privacy considerations are also mandatory before clinical validation is performed. The Artificial Intelligence for Health Imaging (AI4HI) Clinical Working Group has discussed and present in this paper some indicative Machine Learning (ML) enabled decision-support solutions currently under research in the AI4HI projects, as well as the main considerations and requirements that AI solutions should have from a clinical perspective, which can be adopted into clinical practice. If effectively designed, implemented, and validated, cancer imaging AI-supported tools will have the potential to revolutionize the field of precision medicine in oncology.

## Key points

- EU-funded research projects address the creation of AI-supported clinical decision support solutions.
- AI-based models in oncologic imaging need to be fair, robust, and trustworthy.
- Appropriate definition of relevant study parameters is essential to ensure clinical adoption.
- Clinical validation phases of AI-based clinical decision support systems need careful design.

## Background

The Artificial Intelligence for Health Imaging (AI4HI) projects is a network of five EU-funded research projects currently working on Artificial Intelligence (AI) solutions based on medical images and related clinical and molecular data, to improve clinical practice. These projects are Primage (GA 826494), Chaimeleon (GA 952172), EuCanImage (GA 952103), Incisive (GA 952179), and Pro-Cancer-I (GA 952159). Although the projects differ in several key aspects, some common strategies and architectures can be foreseen regarding the efforts to construct validated AI tools using medical imaging and combining with relevant related data to estimate clinical events in daily oncologic practice. Basically, data from electronic health records and PACS is selected and extracted based on defined common data elements, de-identified, harmonized to a common framework, and stored in databases and image repositories before the AI models are trained, tuned, and validated to improve a clinical pathway. In this process, researchers should extract and prepare data (data scientists), construct AI models (AI scientist) and design the study to maximize clinical impact (medical scientist).

**Fig. 1:**
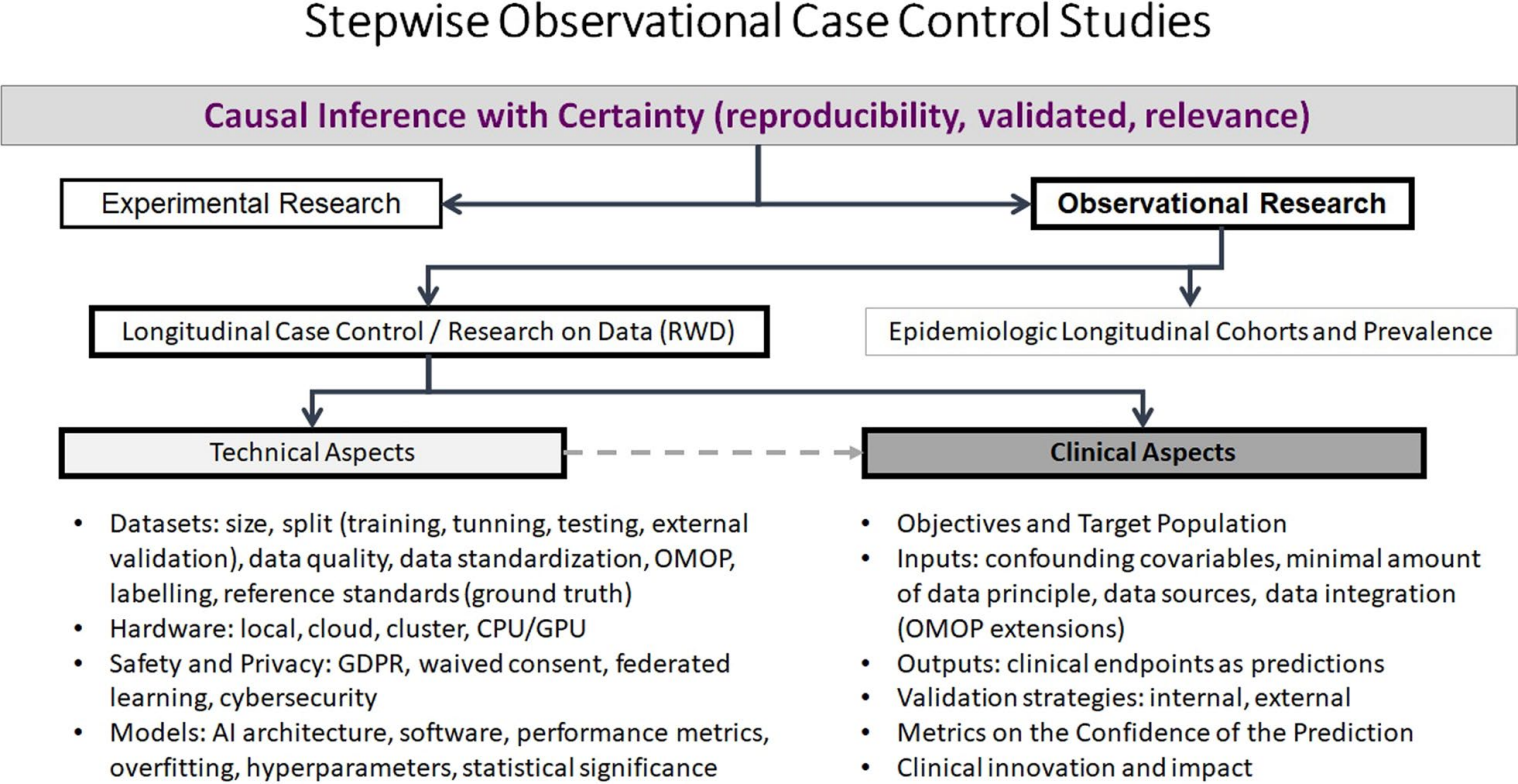
Causality by design: step wise observational case control studies

**Fig. 2:**
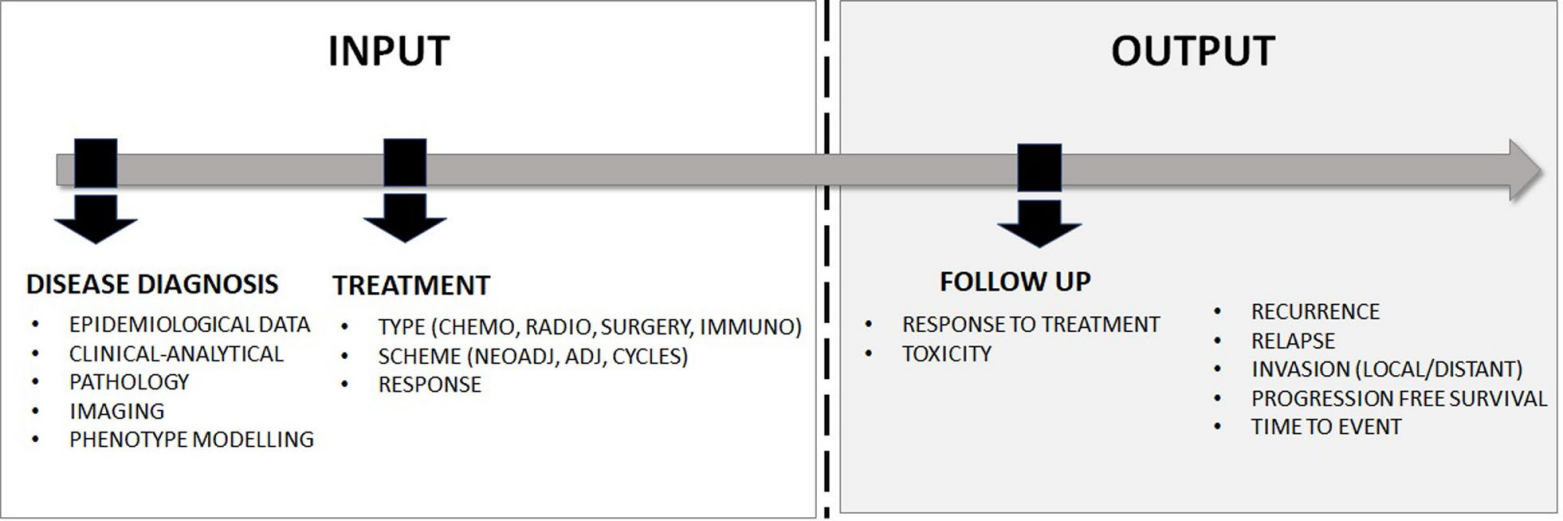
Clinical endpoints (CEPs) and type of data obtained in observational oncology studies

In medical imaging, AI-related research is largely based upon observational, non-interventional in silico studies performed by computer simulation on routinely collected Real World Data (RWD). As the patient episode is usually closed/completed, the dataset in these observational studies is retrospectively collected and anonymized, and there is no possible link between patients, data collection process, and AI-researchers, with such a post hoc study recruitment policy. The non-interventional nature is guaranteed as researchers only address the design, implementation, and evaluation of the AI algorithms in a computational environment (Fig. 1). The data used in these studies come from Electronic Medical Records (EMRs) from the participant hospitals or research biobanks. AI4HI projects are involved in the construction of research repositories as biobanks for cancer images and related data. The created datasets contain use-cases whose collection is defined by the clinical objective, retrieved data, and clinical endpoints (CEPs) of interest (Fig. 2).

**Fig. 3:**
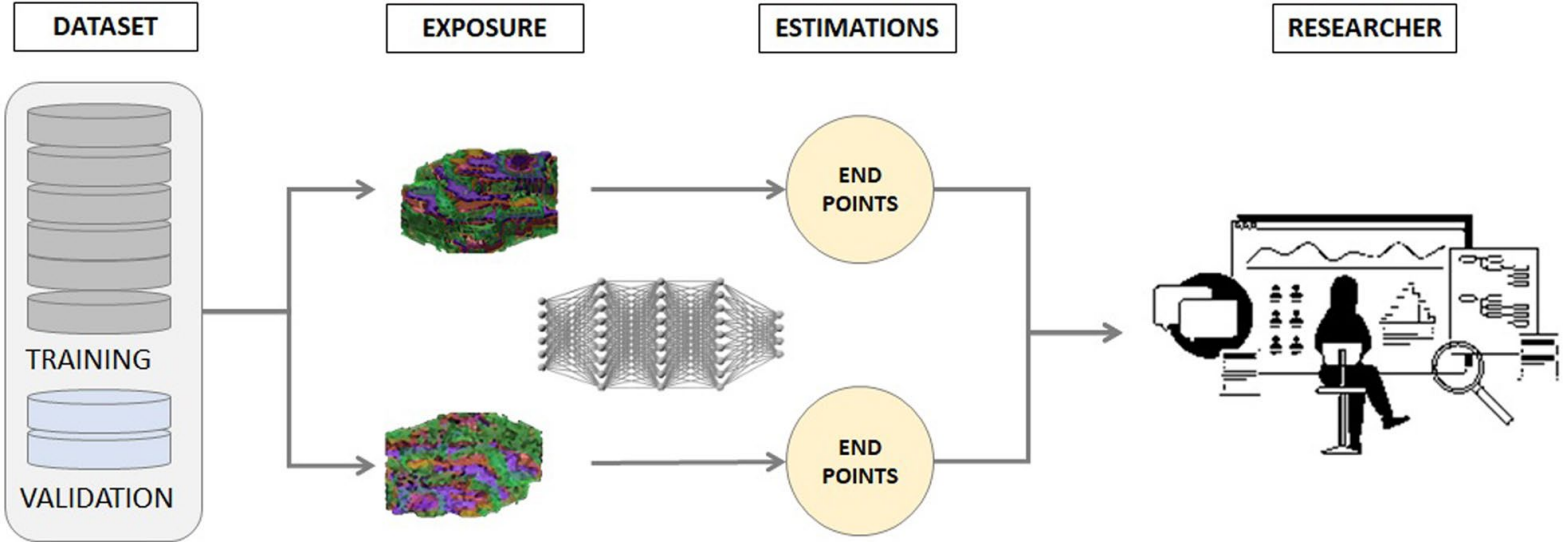
Flow chart from data recruitment and creation of dataset to data visualization

These datasets are used for the training, tuning, and testing of the AI models developed to improve the reproducibility and estimation of CEP events. The training and tuning datasets are used for the construction of the AI solution, while the testing dataset is used for the internal validation analysis (accuracy and repeatability). An external validation set with data from different centers and scanners is constructed and used for a final reproducibility analysis to ensure robustness of the resulting model. The dataset constructed from different centers constitutes the basis for external clinical validations [1] (Fig. 3).

Indicative examples of AI models and Machine Learning (ML) algorithms currently under research and implementation in the AI4HI projects follow. Researchers are developing AI-based models in an open cloud-based platform to support decision-making in the clinical management of two pediatric cancers (neuroblastoma and diffuse intrinsic pontine glioma). The project utilizes standard-of-care MR and CT images at diagnosis and follow-up time points, together with clinical and molecular data, for the prediction of relevant clinical endpoints such as overall survival, time to progression/relapse, and response to treatment. In addition, special emphasis is given to the automation of the image preparation by building image quality control tools based on unsupervised learning techniques (clusterization), creating ML models from DICOM metadata for the labeling of MR sequences, and training convolutional neural networks (CNNs) for the automatic segmentation of tumor and adjacent organs.

Regarding breast cancer, mammographic images are first passed through a ML-enabled classifier trained with both control and abnormal images and related clinical and pathological data. If classified as abnormal, a second classifier is trained to determine the type of abnormality (lesion, calcifications, or both). Depending on the outcome, different AI-based segmentation models look for the respective region of interest and produce the output masks. Additional classification models will be trained to determine the BIRADS score and breast density, features that are of particular importance in the management of patients. The AI4HI ML solutions for breast cancer also address breast MR images to segment and classify the lesions, combining the outputs with other clinical data for precise disease staging.

Other challenges include the development of AI-powered pipelines for data deidentification, curation, annotation, authenticity protection, and image harmonization. In particular, the development of image harmonization Deep Learning (DL) algorithms is based on either Generative Adversarial Networks (GANs), where images from different manufacturers are converted to a reference, and self-supervised learning techniques, where original images and simple transformations are used as input data to a CNN-based autoencoder, which is then trained to reconstruct an harmonized version of the original image.

The application of validated AI-based solutions is essential for precision medicine to provide physicians with a trustworthy clinical decision support system (CDSS) [2]. Having an impact on a specific clinical pathway is defined by the diagnostic gain in comparison with standard of care and the strong relationship between algorithm event predictions and final ground truth. Ensuring several key aspects, such as clearly defining the technical biases and clinical validation phases, and the evaluation of the impact through the strength of the prediction inference is vital for success. To ensure clinical use, the target population, dataset splitting, validation methodology, reference standards, and clinical performance metrics should be clearly identified [3]. Furthermore, CEPs must be carefully selected, whether these are diagnostic disease behavior, treatment response, or patient prognosis or outcome [4–7]. In the field of AI-assisted tools as medical devices, their clinical acceptance requires proven capability of extrapolating the computational solutions into multicentric studies and heterogeneous datasets.

Our objective is to present the main steps for AI research that our AI4HI projects share and envision, including additional desirable validation steps such as largescale external validations which will be mandatory before real-life deployment of the research prototypes. Any developed, validated or deployed AI solution aimed toward specific clinical impact in oncologic imaging must be monitored for the following properties: fairness and unbiasedness, universality and standardization, robustness, reliability, explainability and trustworthiness, traceability and monitoring, as well as usability and equity in transferability [8].

## Objectives and initial considerations

The main general objective of AI-based studies involving cancer imaging data is to provide decision support tools from standard-of-care images and related clinical-molecular data by presenting physicians with estimates or predictions of disease aggressiveness, expected treatment response and final clinical outcome [9]. The data minimization approach should be considered as collected data should not be held unless are essential for the designed study, in accordance with data privacy and legal issues [10–12].

The selection of the target population depends on the primary study objectives and clinical outputs. For the AI models to be generalizable, the selected population should be representative of the clinical disease spectrum and related clinical outputs. Once the target population has been defined and to ensure maximum fairness and universality, it is important to ensure that a sufficiently large sample size is recruited before the prediction models are developed. The required sample size will vary according to several factors, such as the number of predictors (variables) used to characterize the target population, the type of outcomes (continuous, binary or time-to-event) and number of events per variable (e.g., patients in different categories) and the expected predictive performance of the model [13].

Continuous improvement in public health data registries through data digitization and integration with medical images are facilitating the acquisition of real-world data (RWD) in a real-world context. Currently, clinical data, pathological and imaging reports and images are included in EMRs contain a wealth of data that can be consolidated onto ad-hoc custom created data warehouses. After extraction, research data lakes and imaging repositories are created. Unfortunately, most RWD elements are frequently highly unstructured, use non-standard terminologies and lack a common vocabulary, hampering multi-center data harmonization [14]. To partially address this limitation, multicenter projects with complex data specify Common Data Elements (CDEs) models, which contain concise, uniformly structured information stored across different centers that will enable standardized data exchange between different information systems managed at different data providing centers. These CDEs contain standard units and definitions for the clinical data to be registered for the specific clinical targets and endpoints, facilitating the creation of common data repositories that are among the main goals of all the AI4HI projects [15].

The following characteristics might serve as examples for observational, analytical, and in silico predictive studies in oncology:

- Observational nature of the studies. Researchers obtain and document post hoc occurring tumor phenomenon as associations with different outcomes being evaluated (e.g. tumor radiomics for the estimation of overall free survival).
- The researcher does not have any active intervention in the clinical course of the individuals being studied, as the exposure and endpoints have already happened before the start of the data collection.
- The observational study is mainly case–control, where the investigators simply assess the strength of the relationship between exposure to a specific computerized phenotype and a disease endpoint within a temporal dimension [16].
- The characteristics of the subjects, context, exposures, timing, confounders, and interactions are defined before data collection.
- The recruitment and analysis phases are defined as post hoc analyses over known endpoints.
- Data on the relevant events are collected from existing health records and are analyzed once the clinical episode is closed, endpoints are known, and data is de-identified.
- Prediction models are constructed, tested, and validated in silico on datasets from large repositories, linking the multicenter extracted radiomic information with the relevant molecular and clinical data.
- Within repositories, data homogeneity is usually a limiting factor, as structured data warehouses with Common Data Elements (CDEs) standards are not usually available.
- Collected data are used in an aggregated format after careful multilevel (clinical, molecular, imaging) de-identification to ensure patient privacy and to fulfil General Data Protection Regulations (GDPR).
- The data are stored as de-identified cases in imaging repositories where no intervention can be made by the researcher on the patient’s medical history. For legal reasons, tables of ID correspondence are kept at the local level only. The processing of de-identified data is allowed for the purpose of archiving data for public interest, scientific research, or other statistical purposes.
- As research is performed on retrospectively collected RWD, patient informed consent is usually waived by the Ethics Committee at the data provider sites (such is the case in all AI4HI projects). However, patient consent is usually required if data is prospectively collected before the episode is finalized. The access to high-quality large datasets for training and validation is mandatory for clinically relevant AI solutions.

## Checklists for clinically acceptable AI solutions using medical imaging

Some relevant items should be clearly defined in AI studies, which aim to have clinical impact in real world scenarios. These include:

- Well-defined target population. This should cover the whole disease spectrum relevant for the specific questions being predicted. Example: within the Chaimeleon project (evaluating lung cancer), the target population include patients with a diagnosis of non-small cell lung carcinoma who received immunotherapy.
- Adequate sample size calculations. The minimum number of cases required to obtain reliable results, including the optimal balance between healthy and pathological cases needs to be defined [17]. Example: the Chaimeleon project aims to recruit nearly 10,000 prostate patients to enhance the precision and reliability of distinguishing between low-risk from high-risk tumors; and to inform adequate therapy or follow up.
- Standard criteria for clinical and pathological diagnostic considerations. Data dictionary and reference definitions should also be used for treatment response and clinical endpoints. Example: use standardized radiological images as recommended in guidelines. for instance, in breast cancer, bilateral mammography and/or ultrasound of the tumor and lymph node is universal. Lesions are characterized using the BI-RADS classification system (standard Imaging-Reporting and Data System for the breast).
- Specified time points for data collection. Images collected in cancer patients are usually at various time points in the disease journey such as at diagnosis, loco-regional treatment, neoadjuvant therapy, surgery, adjuvant therapy, radiotherapy, relapse or recurrence, last follow-up, and death. The time interval between image acquisitions, diagnosis and treatments should be defined. Example: in the Chaimeleon project, all diagnostic tests must be performed within 2 months of the diagnostic pathology report.
- Minimum amount of data to be collected. To adhere to data minimization principle, only data essential or expected to influence the estimated outcomes under investigation should be collected and integrated. Example: variables directly affecting diagnostic, treatment, or follow-up risk stratification.
- Relevant co-morbidities. Concurrent patient conditions with the studied disease that might have an additional effect on the measured outputs should be included where appropriate. Example: hypertension, diabetes, obesity, and other primary cancers.
- Standards and units for measurements. All quantitative variables and their units should be standardized, choosing the most internationally and frequently used if there are several. Example: use of centimeters or millimeters for tumor size, or Karnofsky performance status for oncological patients in treatment response studies [18].
- Image quality criteria. Before images are incorporated into the de-identified research repository, exclusion criteria based on low image quality must be defined. Standard procedures for data curation and quality control, including protocols addressing poor-quality clinical, pathological, and imaging data submitted to repositories must be defined. Example: several recent solutions have been developed to help interrogate MR datasets, MRQy for variations in resolution or contrast, imaging artifacts such as noise or inhomogeneity [19], or PI-QUAL a prostate-specific tool to assess diagnostic quality of images [20].
- Incorporated source images and extracted data harmonization. To minimize biases associated with different centers, machines, and acquisition protocols, both source images, and extracted data must be normalized to a common framework for reproducibility. Example: specific developed programs such as histogram normalization and discretization [21], ComBat harmonization [22], or Generative Adversarial Networks and unsupervised image-to-image translation units [23, 24].
- Massive data extraction and data interoperability. Example: the use of Observational Medical Outcomes Partnership (OMOP) [25] as the Common Data Model, together with the definition of oncology and imaging extensions is recommended.
- Safety and privacy aspects of repositories. Special focus on de-identification and traceability processes is encouraged. Traceability is generally considered a key requirement for trustworthy AI, being related to “the need to maintain a complete account of the provenance of data, processes, and artifacts involved in the production of an AI model” [26].

## Main variables to be used as inputs to the AI models

The following are common input variables that are used to develop and train AI models:

1. Demographic
  - Age at diagnosis or clinically relevant event: in years and to further detail the time intervals between main diagnostic and therapeutic actions.
  - Gender: biological sex of the patient.
2. Clinical-analytical
  - Tumor staging: standardized descriptions for the amount and spread of the cancer in the patient's body, mainly including tumor size, number, location, vascular invasion, presence of lymph nodes, and presence of distant metastasis.
  - Patient performance status: a score that estimates the patient's ability to perform certain activities in day-to-day life without the help of others. Example: ECOG performance status.
  - Circulating analytical biomarkers: indicators of the biological state or condition that can be accurately and reproducibly measured from either blood, urine, or soft tissue samples. Usually measured to assess the patient status and the responses to a given therapeutic intervention. Example: prostatic specific antigen (PSA) or carcinoembryonic antigen (CEA).
  - Co-morbidities: conditions, other than the primary interest, that the patient has and might influence outcomes. Example: diabetes or arteriosclerosis.
3. Pathology (usually used as referent standard for diagnosis)
  - Tumor type: lesion classification based on cell origin or histological type.
  - Grading: description of a tumor based on how abnormal the cancer cells and tissue are, and how quickly cancer cells are likely to grow and spread.
  - Staging: Description of the extent of the cancer with respect to primary tumor site and size, extent of invasion into local tissues and structures, spread to regional lymph nodes and whether it has metastasized to other regions of the body.
  - Molecular markers: DNA or gene sequence which exact nature and expression levels can be accurately and reproducibly measured.
  - Immunohistochemistry determination: visualization of the distribution and determination of the amount of a given protein in the tissue of interest using antigen–antibody reaction-based detection methods.
  - Mutation profiles: detection of molecular alterations present in a tumor as determined using next-generation sequencing or microarray technologies.
  - Liquid biopsies: non-invasive analyses of circulating tumor-derived material, such as tumor DNA or RNA, tumor cells, extracellular vesicles, or tumor-educated platelets.
4. Imaging
  - Source images: radiological images of different parts of the body used for diagnostic and interventional radiology purposes.
  - Radiomics: quantitative approach used to extract and enhance voxel-wise features from radiographic medical images using data-characterization algorithms [27].
  - Dynamic modeling: workflow that uses time-dependent tomographic images of the same patient, focusing on the changes in image features over time and quantifying them for diagnosis, treatment response or prognostic evaluation.
  - Deep radiomics: use of CNN to analyze and classify texture features from radiological images.
  - Synthetic images or datasets: artificially generated results used for augmentation and enhancement of training sets, as well as for bias prevention (gender distribution, feature distribution) [28].
5. Annotations: either as box (such as bounding box around the malignant tumor), contour/segmentation (such as detailed 3D drawing around the tumor), or points/dots (such as those drawn on the lesion in a mammogram).
6. Treatment Information (needed if a given model is to be trained for treatment response prediction)
  - Surgery: Type of surgery regarding size removed related to the whole organ (e.g., whole mastectomy or tumorectomy), and used instrumentation (e.g., laparoscopic, stereotaxic, cryosurgery, endoscopy)
  - Chemotherapy, immunotherapy, and radiotherapy regimes for response prediction.
  - Sequence of administered treatment options (neoadjuvant, surgery, adjuvant chemotherapy, immunotherapy, radiotherapy).

## Main variables to be defined as outputs for AI predictions

AI solutions must solve specific clinical problems, improve defined clinical pathways, or facilitate targeted clinical decisions. From a clinical perspective, some desired outputs from the AI tools to be prioritized for implementation dealing with oncologic imaging are listed:

1. Phenotyping – Tumor Aggressiveness
  - Growth rate: time at which a tumor volume doubles in size.
  - Direct tumor invasion: invasion of the surrounding stroma by tumoral cells due to loss of cell-to-cell adhesion capacity, changes in cell–matrix interaction that altered cell motility, or acquired migration capacity enabling tumoral cells to invade the surrounding stroma.
  - Lymphatic spread: whether tumoral cells are present in regional lymph nodes near the primary tumor and ultimately, in other parts of the body.
  - Metastasis: when tumoral cells have spread beyond the primary tumor to different parts of the body and the formation of new tumors (secondary and tertiary foci) has occurred. Regional metastasis is that where cells have spread near the primary site, and distant metastasis is defined as that where organs or lymph nodes that are distant from the primary tumor have been affected.
  - Tumor heterogeneity: genetic and phenotypic differences between tumors of the same type in either different patients or within the same patient, and between different cancerous cells within a given tumor.
  - Radiogenomics: correlation, if present, between cancer imaging features and genomics (gene expression patterns, gene mutations and other genome-related characteristics [29].
2. Treatment Response Prediction
  - Response to loco-regional treatment: response to local treatment (usually ablation, embolization or radioembolization) and evaluation of the treatment response after treatment.
  - Response to neoadjuvant treatment: response to systemic treatment administered before surgery in patients without metastasis. It is of great importance to collect when a complete pathological response from other categories (partial, stable response or progression) has occurred, due to its implication in a much better prognosis.
  - Response to adjuvant treatment: response to systemic treatment administered after surgery in patients without metastasis.
  - Response to metastatic treatment: response to systemic treatment administered in patients with metastasis.
  - Response to surgery: response to local treatment and evaluation of the treatment response after surgery.
  - Response to radiotherapy: response to local treatment and evaluation of the treatment response after radiotherapy. Radiotherapy can be used either with curative intent for complete tumor eradication or local control, or with palliative intent to reduce tumor growth and symptom control.
  - Response to novel or targeted therapies (alone or in combination)
  - Side effects and toxicity effects: development of undesired events related to treatment.
3. Clinical Endpoints
  - Downstaging: decrease of the size and extent of primary disease or metastases, and/or lymph node involvement of a tumor by means of anticancer therapy.
  - Tumor regression grading: determination of the amount of residual tumor in patients who underwent preoperative therapy.
  - Status of margins affected: determination of whether residual tumor remains at the surgical resection margins in patients who underwent surgery.
  - Overall survival: time length from either the date of diagnosis or start of cancer treatment to the time of death.
  - Tumor or response-free survival: time length after the patient’s primary treatment without any signs or symptoms of that cancer.
  - Progression-free survival: time length during and after treatment where the disease remains but the patient does not worsen.
  - Time to progression: time length from the date of diagnosis or start of treatment until the disease starts to worsen or spread to other parts of the body.
  - Objective Response Rate: percentage of patients who have a partial or complete response to the treatment within a given time.
  - Complete Response Rate: percentage of patients who have a complete response to the treatment as determined by complete disappearance of lesions within a given time period.

## Clinical validation

The main clinical validation steps that all AI4HI projects will follow deal mainly with models exploring large repositories from real-world data. This section discusses a general clinical validation process that should be considered before the developed AI tool is ready for clinical implementation. The clinical validation of any AI-based CDSS is meant to define the real-life deployment potential of the tool and the extent to which it may impact the daily clinical practice by supporting clinicians to improve the outcome of the patient. The transition from research to clinical practice can be achieved through appropriately planned and conducted studies using internal and external cohorts of patients. Clinical applicability should be promoted by a robust validation across vendor systems, field strengths (for MRI scanners), and institutions [30].

**Fig. 4:**
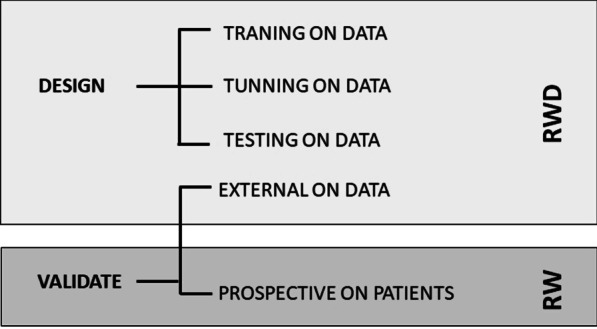
Scheme of the main clinical validation steps in real world data projects

This clinical validation should include both retrospective data and prospective on patient validation steps. In the retrospective validation, the algorithm’s output is validated against independent clinical decisions, reference standards, and/or the ‘ground truth’ [31]. In a prospective validation, one or more clinicians prospectively make clinical decisions having seen the algorithm's output [31–34]. This decision is then validated against independent clinical decisions, investigating for potential introduction of decision bias [32, 35] (Fig. 4). As an example, the prospective validation can evaluate algorithms that produce contours around tumors or other regions of interest (such as for radiotherapy planning), where the output could be deemed ‘good enough’ when prospectively evaluated while differences may be revealed when retrospectively evaluated.

A modified ‘Turing test’ [36] may also be used for further clinical validation. In this process, several outputs from clinical experts and the algorithm under validation are pooled and presented to a blind expert whose task is to identify if the outputs were generated by a colleague or an algorithm [37]. In a similar fashion, the external human reader’s preference for automatic or human output generations can be assessed. Furthermore, this validation might address the effect of AI-based CDSS on clinicians’ decision-making, paying special attention to differences in AI usage by experienced and less experienced clinicians and identifying potential benefits and drawbacks of the integration of AI-based CDSS in clinical practice [38]. Alternatively, a new definition of the intended uses and populations for the assessed AI tools might be necessary [31, 33].

The next step to follow would be an external comparative study in the form of a targeted Controlled Trial of the developed algorithms with the same diagnostic task using large-scale, multicenter, multivendor standardized dataset that the algorithms were not exposed to during previous development phases [31]. There is a question of whether such validation should be initiated and/or funded by the research teams that developed the tool or should there be external incentives to validate newly developed instruments [30]. It is a broad-scale initiative that should preferably be conducted by objective external research teams or organizations affiliated with regulatory bodies on large datasets to ensure the highest-quality external validation of algorithms from different developers.

The external clinical evaluation, apart from standard accuracy assessment with the area under the Receiver-Operating Characteristic (ROC) curves, sensitivity, specificity, false positive and false negative rates, and model’s confidence levels, should also address the influence of the AI tools on the patient’s outcomes [31, 33, 35]. Similar to the drug development process, AI tools should undergo prospectively planned, pre-registered, diagnostic, trials with clearly defined study population characteristics with the patients clinical outcomes as the primary outcome and accuracy metrics as a secondary one [31–33, 35, 39]. Clinical Trials should ensure the involvement of experienced and less experienced physicians to assess the performance of an AI Software as a Medical Device (SaMD) against a ‘reference standard' in real-world data situations. At this stage, different types of bias should be assessed and addressed to ensure proper performance in under-represented sub-groups populations [33, 35].

Finally, there is a question of the longitudinal value of currently used methods of validation in the ‘open’ AI medical decision support tool that continuously learns from the new data (as opposed to the ‘locked’ AI algorithm as defined by the US Federal Drug Administration (FDA) [30, 40]. This ‘open AI algorithm’ approach will require designing the re-evaluation strategy for clinical utility [30]. It would also require the algorithms to be explainable and their decisions to be long-term traceable [31].

## Ethical and usability considerations in clinical applications

Trustworthy, validated, ethically correct, and usable AI solutions are linked to human oversight throughout the process of design, development, evaluation and eventual final practical application and monitoring. There is no doubt that physicians need to be always in control of the clinical decisions, having the first say in matters related to the ways in which AI will support clinical decision-making. Clinicians need to be involved and trained to do this task properly.

There are currently several misconceptions related to the potential, strengths, and weaknesses of AI in clinical practice, such as over- or under-valuing AI tools, overgeneralization of the diagnostic task that the AI algorithm is meant to support, lack of awareness of the strengths and/or limitations of AI tools, unfamiliarity of health care professionals with IT supporting medical practice and difficulty of integrating it in medical practice due to lack of time and resources. Even if the ideal validation framework for AI could be defined and applied to a given AI-based CDSS, AI-related misconceptions could still be the cause of wrong and potentially harmful use of AI in medical practice. This is because there will always be limitations in what AI can achieve and to which extent it can support medical practice. Special emphasis is placed on actions, such as training activities, that target the familiarization of medical experts with AI tools and support them in fully understanding their strengths and limitations. This is the only way to ensure that humans are always in control and that the full potential of AI tools is utilized for making informed decisions.

Currently, the question of accountability when an AI-based system is deployed in real clinical settings and either fails or produce unexpected outcomes is still open and burning [41]. The problem affects any algorithmic application that supports decision-making, being debated in the ethic, social and legal communities [42].

Another aspect requiring attention and further work in conjunction with ethical and legal experts is the situation of diagnosing new health issues in a subset of the investigated cohort that was not diagnosed before, due to the lack of appropriate tools. Due to technical issues of de-identification, the AI researcher on data should have no direct responsibility towards patients and the only foreseeable solution is to inform the responsible physician. There might be several clinical and legal issues in such a situation, such as (1) the patient died of the undiagnosed condition, (2) the disease progressed without being detected, (3) the disease progressed due to late detection, or (4) the disease failed to be treated due to the late detection. These aspects require elaboration while the AI algorithms become more precise and sensitive, such as the tool capable of predicting future breast cancer based on subtle image features [43]. These clinical issues concern physicians and their obligation to deliver the best possible care for their patients.

Another important ethical consideration is related to possible limitations of the training dataset that will be used for AI training, which must represent various demographics to the best possible extent to avoid inherent bias [44]. When this is not feasible, vendors should clearly inform clinical users of potential biases towards gender, ethnicity, age, or any other disparities. Practitioners using AI algorithms in clinical practice would need to seek such information and make sure they consider any inherent bias of the AI algorithm during the interpretation of its outcome.

Clarity of the design and biases control are extremely important items to report when releasing an AI solution with medical data and images. Some of the most relevant aspects to check include:

- The scientific background regarding the clinical problem.
- The study design regarding target population and study sample size.
- Patient recruitment and data extraction.
- Data quality analysis.
- Data curation and image preparation.
- Data anonymization.
- Data annotation.
- Dataset partitioning.
- Reference standard definitions.
- AI models, training procedures, and hyperparameters.
- Metrics used for validation.
- Model robustness and explainability.
- Proposed use in daily clinical practice.

## Conclusions

When designing an AI-based research study in oncologic imaging, the proper definition of several key aspects is essential to ensure the highest possible impact in current clinical practice. These include factors related to the right selection of the target population, sample size, clinical endpoints and proper definition of clinical variables to be used both as input and output to the AI models, ensuring safety and patient privacy to fulfill GDPR. AI-assisted medical solutions need to be robust and trustworthy, with well-designed and performed clinical validation phases. In this article, we have discussed the importance of several key aspects related to AI-based studies in oncologic imaging, providing clear definitions to the usual type of studies performed and a general checklist to be followed when executing both real-world data and real-world validation phases to have a final impact in precision medical oncology [45].

## Data Availability

Not applicable.

## Abbreviations

CDE: Common data elements
EMR: Electronic medical records
FDA: Federal drug administration (USA)
GDPR: General data protection regulations
OMOP: Observational medical outcomes partnership
RWD: Real world data
SaMD: Software as a medical device
